# Visual and digital assessment of Ki-67 in breast cancer tissue - a comparison of methods

**DOI:** 10.1186/s13000-022-01225-4

**Published:** 2022-05-06

**Authors:** Anette H. Skjervold, Henrik Sahlin Pettersen, Marit Valla, Signe Opdahl, Anna M. Bofin

**Affiliations:** 1 Department of Clinical and Molecular Medicine, Faculty of Medicine and Health Sciences, Norwegian University of Science and Technology, Erling Skjalgssons gate 1, Trondheim, Norway; 2 Department of Pathology, St. Olav’s Hospital, Trondheim University Hospital, Trondheim, Norway; 3 Department of Public Health and Nursing, Faculty of Medicine and Health Sciences, Norwegian University of Science and Technology, Trondheim, Norway

**Keywords:** Ki-67, Cell proliferation, Immunohistochemistry, Digital pathology, Digital image assessment, Breast cancer

## Abstract

**Background:**

In breast cancer (BC) Ki-67 cut-off levels, counting methods and inter- and intraobserver variation are still unresolved. To reduce inter-laboratory differences, it has been proposed that cut-off levels for Ki-67 should be determined based on the in-house median of 500 counted tumour cell nuclei. Digital image analysis (DIA) has been proposed as a means to standardize assessment of Ki-67 staining in tumour tissue. In this study we compared digital and visual assessment (VA) of Ki-67 protein expression levels in full-face sections from a consecutive series of BCs. The aim was to identify the number of tumour cells necessary to count in order to reflect the growth potential of a given tumour in both methods, as measured by tumour grade, mitotic count and patient outcome.

**Methods:**

A series of whole sections from 248 invasive carcinomas of no special type were immunohistochemically stained for Ki-67 and then assessed by VA and DIA. Five 100-cell increments were counted in hot spot areas using both VA and DIA. The median numbers of Ki-67 positive tumour cells were used to calculate cut-off levels for Low, Intermediate and High Ki-67 protein expression in both methods.

**Results:**

We found that the percentage of Ki-67 positive tumour cells was higher in DIA compared to VA (medians after 500 tumour cells counted were 22.3% for VA and 30% for DIA). While the median Ki-67% values remained largely unchanged across the 100-cell increments for VA, median values were highest in the first 1-200 cells counted using DIA. We also found that the DIA100 High group identified the largest proportion of histopathological grade 3 tumours 70/101 (69.3%).

**Conclusions:**

We show that assessment of Ki-67 in breast tumours using DIA identifies a greater proportion of cases with high Ki-67 levels compared to VA of the same tumours. Furthermore, we show that diagnostic cut-off levels should be calibrated appropriately on the introduction of new methodology.

## Introduction

Sustained proliferative signalling is one of the hallmarks of cancer, as proposed by Hanahan and Weinberg in 2011 [1]. The nuclear antigen detected by the Ki-67 antibody is a marker of the growth fraction of a tumour. It is expressed in the G1, S, G2 and M phases of the cell cycle, but not in the resting phase, G0. While expression levels are low in G1 and S, they peak during G2 and M [2]. In breast cancer (BC), immunohistochemical (IHC) staining of the Ki-67 antigen is commonly used in the assessment of the proliferative activity of the tumour. It can provide information on prognosis and predict response to treatment in the adjuvant and neoadjuvant settings [3–6]. High Ki-67 score is associated with poor prognosis [7] but also a good response to chemotherapy [8, 9].

In molecular subtyping of BC, Ki-67 can be used to distinguish between Luminal A-like (Ki-67 low) and HER2 negative Luminal B-like (Ki-67 high) BC subtypes [10, 11]. While Luminal A patients generally have a good prognosis and may qualify for endocrine treatment only, Luminal B patients have a poorer prognosis and will often be given chemotherapy in addition. Thus, differentiation between these two subtypes has important therapeutic value [8, 10, 12].

Although the clinical validity of the Ki-67 Proliferation Index is accepted in BC, its clinical utility is still regarded as limited and there is a lack of consensus on the appropriate number of cells to count and cut-off levels for prognostication and treatment [13]. Furthermore, inter- and intra-observer agreement in the assessment of Ki-67 is poor [14–19].

Ki-67-staining is often heterogeneous within a tumour [20, 21]. In the assessment of Ki-67 IHC, only positively stained nuclei and mitotic figures should be scored, regardless of staining intensity, and between 500 and 1000 tumour cells should be counted in hotspot areas [22, 23]. According to the International Ki67 in Breast Cancer Working Group, Ki-67 levels between 5% and 30% are subject to considerable interobserver and interlaboratory variability. They suggest that only very low (< 5%) or very high (≥ 30) levels should be considered clinically actionable [13, 24]. To ameliorate issues of inter-laboratory variation, the 14th St. Gallen International Breast Cancer Conference in 2015 proposed that the in-house median value at each laboratory should be used to determine cut-off values due to interlaboratory differences [17].

Several studies have suggested the use of automated digital image analysis (DIA) to improve reproducibility in the assessment of Ki-67. With the introduction of DIA, it should be possible to redefine interpretation algorithms for biomarker assessment for both established clinical and novel biomarkers in BC, and address the issue of inter- and intraobserver variation in the interpretation of these biomarkers [15, 18, 19, 25–29].

In this study we compared visual assessment (VA) and DIA of tissue sections stained for Ki-67 in a consecutive series of BCs. The aim was to identify the number of tumour cells necessary to count in each method to reflect the growth potential of a given tumour, as measured by tumour grade, mitotic count and patient outcome.

## Materials and methods

### Study population

The study comprises 250 BCs from a larger series of BC patients. The background population from which this series arises comprises 25,727 women born between 1886 and 1928 in Nord-Trøndelag County in Norway, who were followed for BC occurrence from 1961 to 2008. In total, 1379 cases of BC were diagnosed during follow-up, and 909 of these tumours were classified into six molecular subtypes using IHC and chromogenic *in situ* hybridization (CISH) as surrogates for gene expression analysis [30]. After diagnosis, all patients were followed until death from BC, or death from other causes or until December 31st, 2015 [30, 31].

**Fig. 1:**
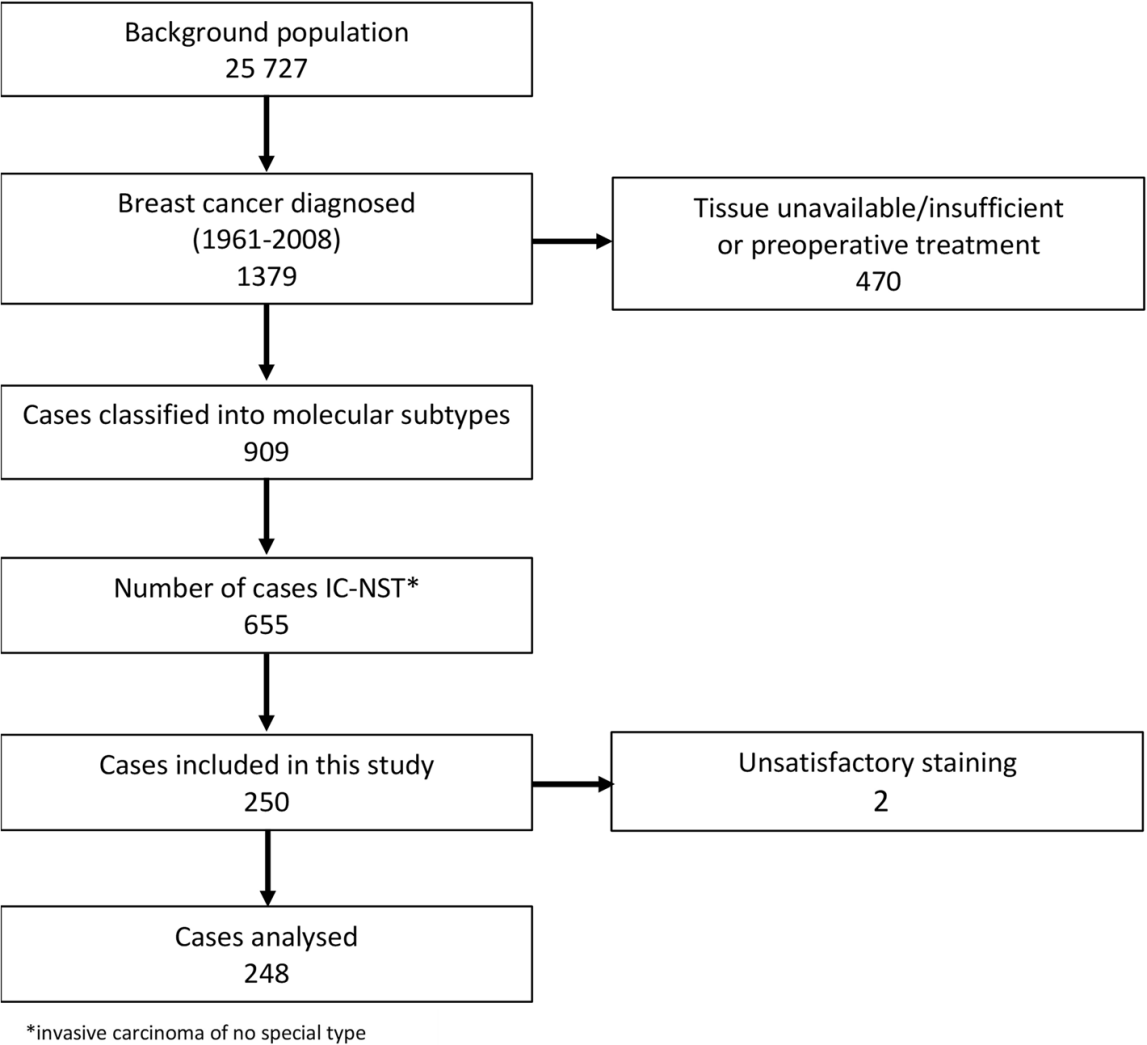
Flowchart showing an overview of the cases included in this study

In the present study, we included 250 consecutive cases of invasive carcinoma of no special type [32]. Two cases were excluded due to unsatisfactory staining (Fig. 1).

### Immunohistochemistry

Full-face sections 4 μm thick, mounted on SuperFrost glass slides, were retrieved from storage (-20 °C). Paraffin was removed using TissueClear and sections were rehydrated with ethanol and water. Slides were heated at 60 °C for two hours and pretreated in a PT Link Pre-Treatment Module for Tissue Specimens (Dako Denmark A/S, 2600 Glostrup, DK) with a buffer (Low pH Target Retrieval Solution K8005) at 97 °C for 20 min. The Ki-67 antibody was applied (Clone MIB1, 35 mg/L, 1:100, Dako Denmark A/S, Glostrup, Denmark) in a DakoCytomation Autostainer Plus (Dako), with 40 min incubation time. Dako REAL™EnVision™ Detection System with Peroxidase/DAB+, Rabbit/Mouse (K5007), was used for visualization.

### Digital image analysis

The IHC-stained slides were scanned at 40X magnification with a resolution of 0.23 μm/pixel using Hamamatsu NanoZoomer S360 Digital Slide scanner C13220-01 (Inter Instruments AS) at the Department of Pathology, St. Olav’s Hospital, Trondheim University Hospital, Norway. The digital images were analysed for Ki-67 protein expression using the open-source, DIA software QuPath v. 0.1.2 [27].

#### Training of the classifier

A separate series of 19 representative cases from the main cohort were used as a training set to train a two-class object classifier in QuPath after watershed nucleus detection [27]. The tumour area was delineated manually in the QuPath software. Cell nuclei (training objects) were selected and defined as either epithelial tumour cell nuclei or other (non-tumour cell nuclei or tumor stroma cell nuclei) in the whole slide images (WSI).

In the training set, stains were digitally separated using the colour deconvolution method and the automated “Estimate stain vectors” function in QuPath [27]. Watershed cell nucleus detection was performed and optimized visually using the following settings: Optical density (OD) sum; requested pixel size 0.4 μm; background radius 8.0 μm; median filter radius 1.5 μm; sigma 1.5 μm; min/max area 10/350 µm; threshold 0.02; maximum background intensity 3.0; and cell expansion 5 μm. Smoothing of object features (25, 50 and 100 μm) was applied. The threshold value for Ki-67-positivity (nucleus DAB OD mean) was assessed and adjusted manually, to best correspond to the visual perception of Ki-67 positivity in VA. Hence, the threshold was finally set to 0.15 nucleus DAB OD mean for all slides.

A cell nucleus detection object two-class Random Trees classifier (tumour cell nuclei vs. non-tumour cell nuclei) was trained using the default settings [27]. Training continued until visibly acceptable classification was achieved using 67% equally spaced train/test-split, resulting in approximately 85% accuracy. This was obtained using 7514 training objects and 135 object features from the 19 annotated images in the training set. The classifier was saved and applied to the watershed nucleus detections within the manually annotated tumor areas of all 248 cases in this study.

**Fig. 2:**
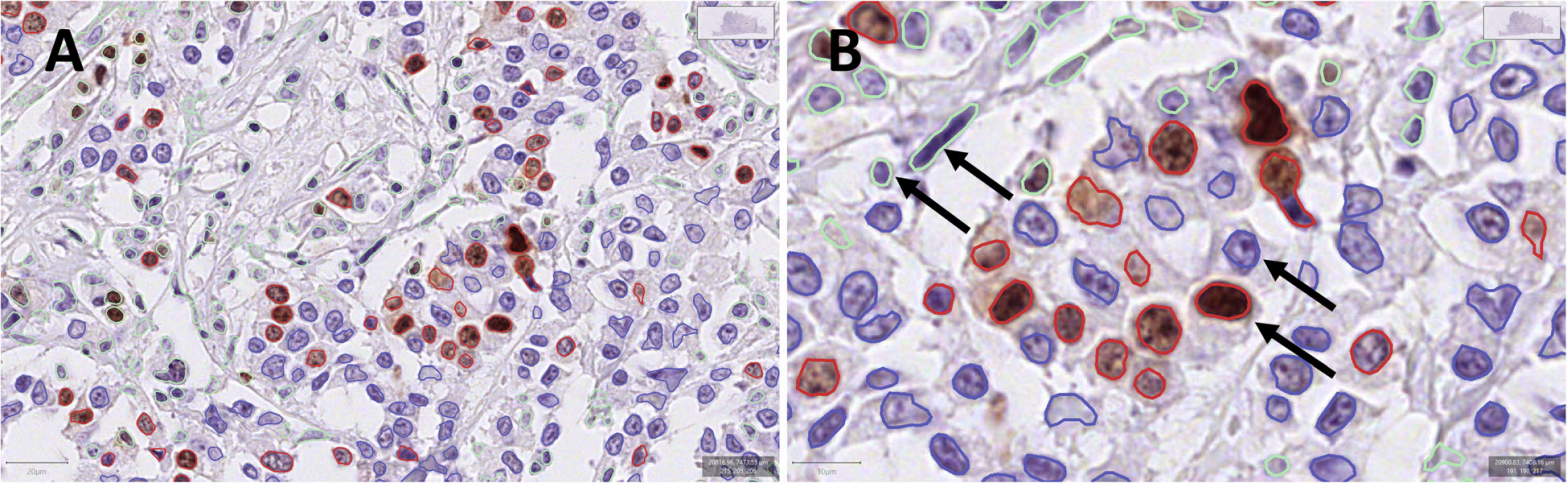
**A** Overview image from QuPath showing cell nucleus detection and classification. **B** Arrows indicate elongated stromal nucleus and lymphocyte (green); Ki-67 positive tumour cell nucleus (red) and Ki-67 negative tumour cell nucleus (blue)

All nuclei in the tumour were detected by running positive cell nucleus detection provided by QuPath, and then sub-classified into epithelial tumour cell nuclei and other intra-tumoural nuclei by the trained classifier. Due to the heterogeneity of BC tissue, additional annotations were subsequently added to the classifier for most of the digital images until visually acceptable discrimination between epithelial tumour cell nuclei and all other nuclei was achieved for each WSI. Examples of annotation of training objects are shown in Fig. 2.

#### Digital Ki-67 hotspot identification

**Fig. 3:**
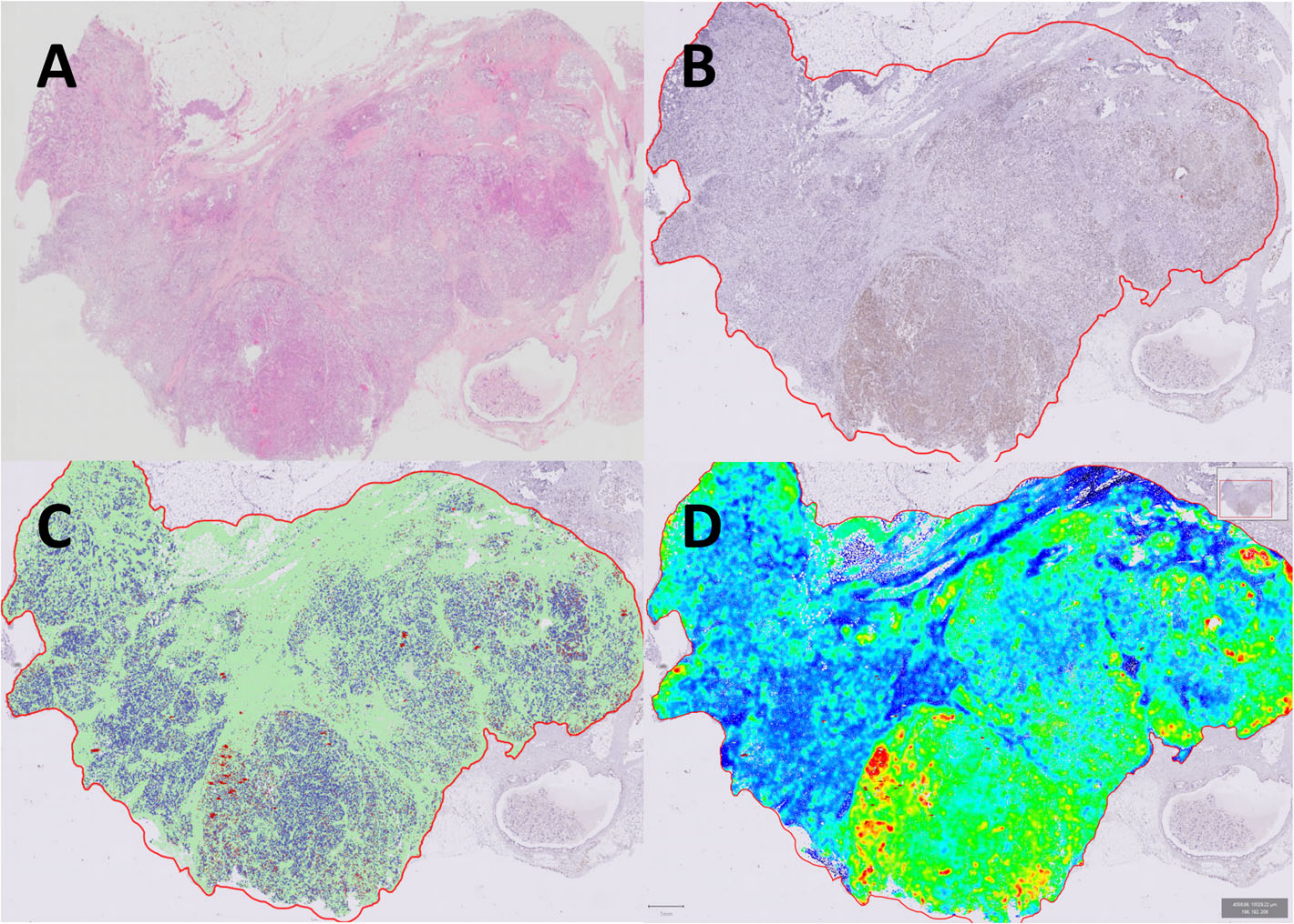
**A** HES stained WSI; **B** IHC stained WSI (Ki-67) with manually delineated tumour area (red); **C** Cell detection within the tumor area and tumor (blue/red) and non-tumor (green) classified cells; **D** Measurement heat map showing Ki-67 hot-spots in red

The tumour area in each of the 248 full-face sections was delineated manually by an experienced breast pathologist and the manual delineation was thereafter used to guide digital delineation of the tumour in the WSIs in the QuPath software. Ki-67 positive tumour hotspot areas were identified using a semi-automated approach by generating measurement heat maps in QuPath by visualizing nucleus DAB OD mean: Smoothed 50 μm. The heat maps were manually adjusted for each WSI to identify and annotate the area with the highest density of Ki-67 positive tumour cell nuclei (Fig. 3A-D). Areas with obvious artefacts resulting in false hotspots were manually excluded.

### Scoring and reporting

#### Visual assessment

Visual assessment of Ki-67 proliferation rate was done using a brightfield microscope (Nikon Eclipse 80*i*) at 40x magnification. A total of 500 tumour cell nuclei (5 × 100) were counted in visually selected hotspot areas in each case, starting with the group of 100 cell nuclei which appeared to have the highest proportion of Ki-67 positive cells. The number of positive-staining tumour cell nuclei was recorded separately for each 100-cell increment counted.

#### Digital image analysis

**Fig. 4:**
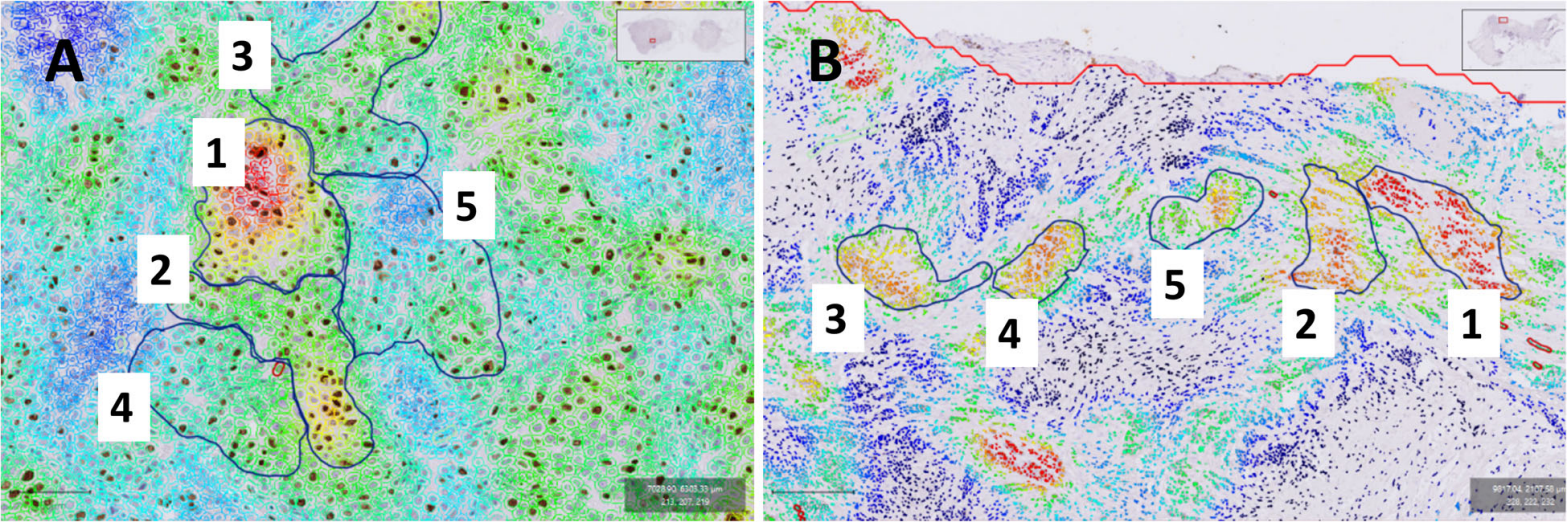
**A **and** B** Hotspot identification and delineation images from QuPath. Areas of 100 tumour cell nuclei ordered from the area with the highest proportion of Ki-67 positive tumour cell nuclei [1] to the lowest [5]

All cases were assessed for Ki-67 expression using the QuPath software. Once the Ki-67 tumor hotspot was identified using the measurement heat map, five areas containing 100 tumour cell nuclei were manually delineated using the QuPath “brush tool”. Counting started in the group of 100 nuclei that, within the identified hotspot, appeared to have the highest density of positive staining nuclei according to the heat map and continued in decreasing order of density until five sets of 100 nuclei were counted (Fig. 4).

#### Cut-off levels for Ki-67 Low/Intermediate/High positivity

**Fig. 5:**
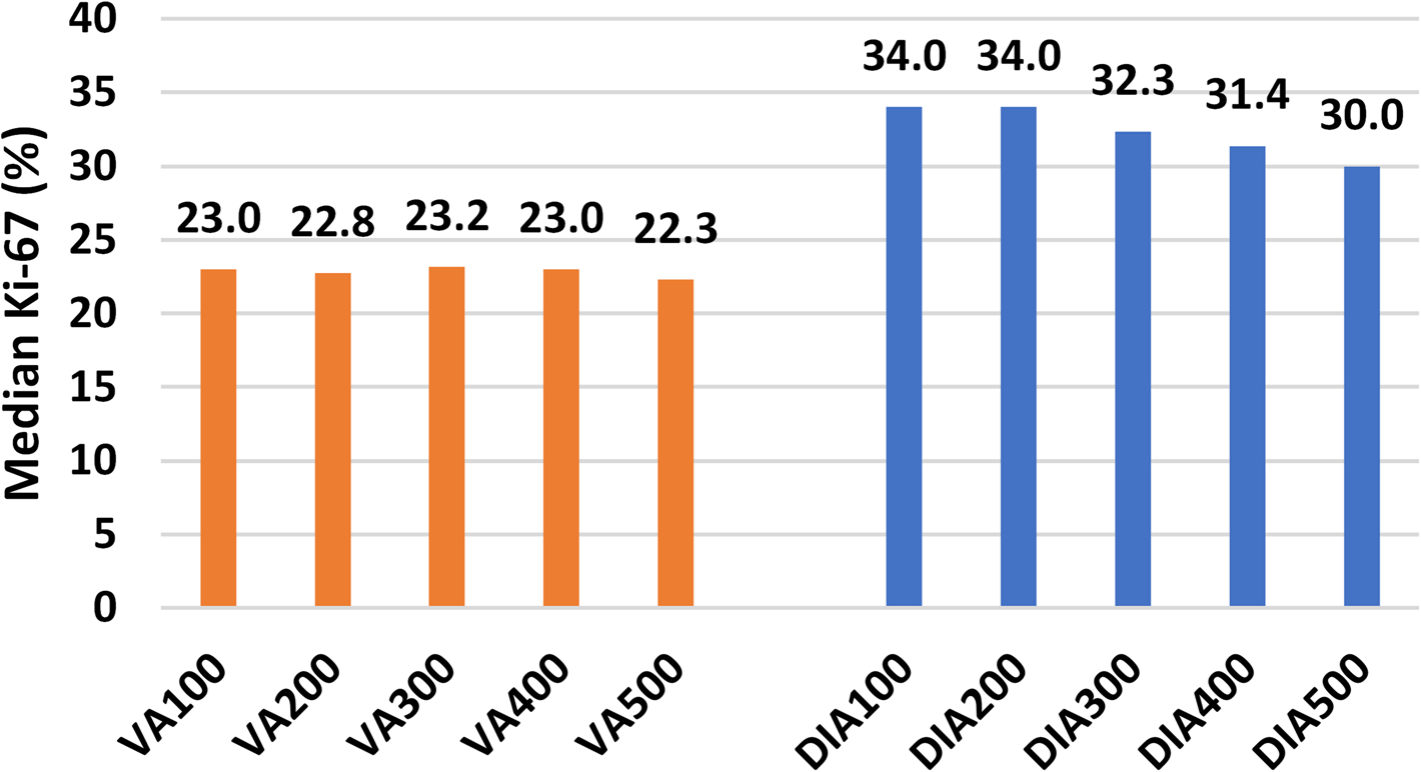
Median Ki-67 at each cumulative 100-cell increment for visual assessment (VA) and digital image analysis (DIA)

We determined cut-off levels based on the median Ki-67 values for each method according to the St. Gallen International Expert Consensus on the Primary Therapy of Early Breast Cancer 2015 [17]. Ki-67 Low was defined as 10% points below the median, and Ki-67 High as 10% points above the median. Values falling between Low and High were classified as Intermediate. The median values of Ki-67 positivity using VA and DIA were calculated for 100 cells (VA100, DIA100); 200 cells (VA200, DIA200); 300 cells: (VA300, DIA300); 400 cells (VA400, DIA400); and 500 cells (VA500, DIA500) (Fig. 5). In the statistical analyses, only the results for VA/DIA100 and VA/DIA500 were used.

### Statistical analyses

Tumour characteristics were compared using Pearson’s Chi squared test across categories of VA and DIA (Low, Intermediate and High as described above) for 100 and 500 nuclei counted. Bland-Altman plots were used to evaluate the agreement between VA500 as the reference measurement, and DIA100 and DIA500, by estimating the difference between the methods in relation to the mean. Cumulative incidence of death from BC was calculated for VA100, VA500, DIA100 and DIA500, treating death from other causes as competing events. Gray´s test was used to compare equality between cumulative incidence curves. Cox proportional hazard analyses were used to estimate hazard ratios (HR) of BC death, with censoring at death from other causes. Harrell’s C-test was used to compare the predictive ability of VA100, VA500, DIA100 and DIA500. All analyses were performed using Stata v. 16.0 (StataCorp LP, College Station, Texas, USA).

## Results

Patient and tumour characteristics are presented in Table 1. Of the 248 patients evaluated in this study, 108 had died of BC and 124 had died of other causes by the end of follow-up. There were 16 (6.5%) histopathological grade 1, 131 (52.8%) grade 2, and 101 (40.7%) grade 3 tumours.

**Table 1:** Patient and tumour characteristics according to Ki67 visual assessment (VA) and digital image analysis (DIA) of full face tissue sections

Total study population		VA categories 100 cells (median 22.3%)				VA Categories 500 cells (Median 22.3%)				DIA Categories 100 cells (Median 30%)				DIA Categories 500 cells (Median 30%)			
		**< 12.3**	**> 12.3-< 32.3**	**> 32.3**	**χ**^**2**^	**≤ 12.3**	**> 12.3- < 32.3**	**≥ 32.3**	**χ**^**2**^	**≤ 20**	**> 20-< 40**	**≥ 40**	**χ**^**2**^	**≤ 20**	**> 20-< 40**	**≥ 40**	**χ**^**2**^
N (%)	248	48	123	77		44	132	72		44	104	100		75	94	79	
Mean age at diagnosis range (42–95) (SD)	69.9 (10.9)	69.7 (11.0)	69.8 (11.5)	70.2 (9.9)		71.7 (11.3)	69.0 (10.9)	70.6 (10.5)		71.0 (10.5)	69.4 (11.2)	69.9 (10.8)		70.5 (11.0)	69.4 (11.2)	70.0 (10.5)	
Mean follow-up, years (SD)	10.9 (9.6)	12.3 (10.3)	11.3 (9.6)	9.4 (9.1)		12.6 (10.4)	11.4 (9.5)	8.9 (9.1)		11.7 (9.4)	11.5 (10.1)	9.9 (9.1)		11.0 (10.0)	12.1 (9.5)	9.4 (9.3)	
Deaths from breast cancer (%)	108 (43.6)	19 (39.6)	46 (37.4)	43 (55.8)	< 0.001	17 (38.6)	49 (37.1)	42 (58.3)	< 0.001	15 (34.1)	39 (37.5)	54 (54.0)	< 0.001	28 (37.3)	38 (40.4)	42 (53.2)	< 0.001
Deaths from other causes (%)	124 (50)	25 (52.1)	69 (56.1)	30 (39.0)		22 (50.0)	75 (56.8)	27 (37.5)		25 (56.8)	57 (54.8)	42 (42.0)		41 (54.7)	49 (52.1)	34 (43.0)	
**Histologic grade (%)**																	
I	16 (6.5)	6 (12.5)	10 (8.1)	0	< 0.001	6 (13.6)	10 (7.6)	0	< 0.001	5 (11.4)	11 (10.6))	0	< 0.001	9 (12.0)	7 (7.5)	0	< 0.001
II	131 (52.8)	38 (79.2)	72 (58.5)	21 (27.3)		34 (77.3)	84 (63.6)	13 (18.1)		34 (77.3)	67 (64.4)	30 (30.0)		54 (72.0)	56 (59.6)	21 (26.6)	
III	101 (40.7)	4 (8.3)	41 (33.3)	56 (72.7)		4 (9.1)	38 (28.8)	59 (82)		5 (11.4)	26 (25.0)	70 (70.0)		12 (16.0)	31 (33.0)	58 (73.4)	
**Lymph node metastasis (%)**																	
Yes	96 (38.7)	18 (37.5)	44 (35.8)	34 (44.2)	0.274	17 (38.6)	47 (35.6)	32 (44.4)	0.536	18 (41.0)	35 (33.7)	43 (43.0)	0.630	28 (37.3)	31 (33.0)	37 (46.8)	0.135
No	96 (38.7)	19 (39.6)	53 (43.1)	24 (31.2)		18 (40.9)	53 (40.2)	25 (34.7)		18 (41.0)	41 (33.7)	37 (37.0)		29 (38.7)	42 (44.7)	25 (31.7)	
Unknown histology	56 (22.6)	11 (22.9)	26 (21.1)	19 (24.7)		9 (20.5)	32 (24.2)	15 (20.8)		8 (18.2)	28 (27.0)	20 (20.0)		18 (24.0)	21 (22.3)	17 (21.5)	
**Tumor size (%)**																	
≤ 2 cm	113 (45.6)	19 (39.6)	65 (52.9)	29 (37.7)	0.317	17 (38.6)	70 (53.0)	26 (36.1)	0.162	21 (47.7)	52 (50.0)	40 (40.0)	0.863	33 (44.0)	48 (51.1)	32 (40.5)	0.562
> 2-≤ 5 cm	34 (13.7)	6 (12.5)	15 (12.2)	13 (16.9)		5 (11.4)	14 (10.6)	15 (20.8)		6 (13.6)	12 (11.5)	16 (16.0)		8 (10.7)	15 (16.0)	11 (13.9)	
Uncertain, but > 2 cm	30 (12.1)	5 (10.4)	15 (12.2)	10 (13.0)		7 (15.9)	13 (9.9)	10 (12.1)		5 (11.4)	11 (10.6)	14 (14.0)		9 (12.0)	11 (11.7)	10 (12.7)	
Uncertain	71 (28.6)	18 (37.5)	28 (22.8)	25 (32.5)		15 (34.1)	35 (26.5)	21 (29.2)		12 (27.3)	29 (27.9)	30 (30.0)		25 (33.3)	20 (21.3)	26 (32.9)	
**Stage (%)**																	
I	114 (46.0)	19 (39.6)	66 (53.7)	29 (37.7)	0.061	18 (40.9)	69 (52.3)	27 (37.5)	0.288	20 (45.5)	52 (50.0)	42 (42.0)	0.599	33 (44.0)	51 (54.3)	30 (38.0)	0.071
II	101 (40.7)	20 (41.7)	46 (37.4)	35 (45.5)		20 (45.5)	48 (36.4)	33 (45.8)		20 (45.5)	39 (37.5)	42 (42.0)		32 (42.7)	35 (37.2)	34 (43.0)	
III	17 (6.9)	4 (8.3)	8 (6.5)	5 (6.5)		4 (9.1)	8 (6.1)	5 (6.9)		2 (4.6)	7 (6.7)	8 (8.0)		7 (9.3)	2 (2.1)	8 (10.1)	
IV	13 (5.2)	4 (8.3)	1 (0.8)	8 (10.4)		1 (2.3)	5 (3.8)	7 (9.7)		1 (2.3)	4 (3.9)	8 (8.0)		1 (1.3)	5 (5.3)	7 (8.9)	
Unknown	3 (1.2)	1 (2.1)	2 (1.6)	0		1 (2.3)	2 (1.5)	0		1 (2.3)	2 (1.9)	0		2 (2.7)	1 (1.1)	0	
**Molecular subtype (%)**																	
Luminal A	110 (44.4)	36 (75.0)	63 (51.2)	11 (14.3)	< 0.001	36 (81.8)	68 (51.5)	6 (8.3)	< 0.001	36 (81.8)	55 (52.9)	19 (19.0)	< 0.001	60 (80.0)	41 (43.6)	9 (11.4)	< 0.001
Luminal B (HER2-)	82 (33.1)	6 (12.5)	40 (32.5)	36 (46.8)		3 (6.8)	45 (34.1)	34 (47.2)		2 (4.6)	29 (27.9)	51 (51.0)		4 (5.3)	37 (39.4)	41 (51.9)	
Luminal B (HER2+)	28 (11.3)	5 (10.4)	11 (8.9)	12 (15.6)		2 (4.6)	14 (10.6)	12 (16.7)		4 (9.1)	11 (10.6)	13 (13.0)		6 (8.0)	9 (9.6)	13 (16.5)	
HER2 type	12 (4.8)	0	5 (4.1)	7 (9.1)		0	3 (2.3)	9 (12.5)		0	6 (5.8)	6 (6.0)		1 (1.3)	5 (5.3)	6 (7.6)	
TN	16 (6.4)	1 (2.1)	4 (3.2)	11 (14.3)		3 (6.9)	2 (1.5)	11 (15.3)		2 (4.6)	3 (3.0)	11 (11.0)		4 (5.4)	2 (2.2)	10 (12.6)	
**Ki67 TMA high/low (%)**																	
Ki67 < 15%	125 (50.4)	41 (85.4)	70 (56.9)	14 (18.2)	< 0.001	41 (93.2)	75 (56.8)	9 (12.5)	< 0.001	42 (95.5)	62 (59.6)	21 (21.0)	< 0.001	69 (92.0)	45 (47.9)	11 (13.9)	< 0.001
Ki67 ≥ 15%	123 (49.6)	7 (14.6)	53 (43.1)	63 (81.8)		3 (6.8)	57 (43.2)	63 (87.5)		2 (4.6)	42 (40.4)	79 (79.0)		6 (8.0)	49 (52.1)	68 (86.1)	
**Mitoses/10 HPF median (IQR p25, p75)** p25 = 3 p50 = 8 p75 = 14.5																	
**Mitoses/10 HPF quartiles (%)**																	
≤ 3	72 (29.0)	33 (68.8)	35 (28.5)	4 (5.2)	< 0.001	29 (65.9)	41 (31.1)	2 (2.8)	< 0.001	26 (59.1)	36 (34.6)	10 (10.0)	< 0.001	44 (57.9)	21 (22.6)	7 (8.9)	< 0.001
> 3-≤8	67 (27.0)	9 (18.8)	49 (39.8)	9 (11.7)		11 (25.0)	49 (37.1)	7 (9.7)		11 (25.0)	41 (39.4)	15 (15.0)		21 (27.6)	38 (40.9)	8 (10.1)	
> 8-≤14.5	47 (19.0)	6 (12.5)	19 (15.5)	22 (28.6)		4 (9.1)	23 (17.4)	20 (27.8)		6 (13.6)	16 (15.4)	25 (25.0)		10 (13.2)	17 (18.3)	20 (25.3)	
> 14.5	62 (25.0)	0	20 (16.3)	42 (54.6)		0	19 (14.4)	43 (59.7)		1 (2.3)	11 (10.6)	50 (50.0)		1 (1.3)	17 (18.3)	44 (55.7)	

*Abbreviations*: *SD *standard deviation, *HER2* human epidermal growth factor receptor 2, *TN* triple negative phenotype, *TMA* tissue microarray, *HPF* high power fields

### Cut-off levels for Low/Intermediate/High Ki-67 positivity

Cut-off levels for Ki-67 positivity were calculated for both VA and DIA according to the median Ki-67 values after 500 tumour cell nuclei were counted (VA500, DIA500). The median Ki-67 level was 22.3% for VA500 and 30.0% for DIA500, as shown in Fig. 5. Thus, for the present study, cut-off levels for VA were set at < 12.3% (Ki-67 Low), ≥ 12.3 ≤ 32.3% (Ki-67 Intermediate) and > 32.3% (Ki-67 High). For DIA, cut-off levels were set at < 20.0% (Ki-67 Low), ≥ 20.0 ≤ 40.0% (Ki-67 Intermediate) and > 40.0% (Ki-67 High).

In VA, there was no clear difference between the median values of the five cumulative 100-cell increments (VA100-VA500) (range 22.3-23.2%). Using DIA, the median value for both DIA100 and DIA200 was 34.0%, falling to 30.0% at DIA500. Cumulative median values for all 100-cell increments (both VA and DIA) are shown in Fig. 5.

#### Visual assessment

**Fig. 6:**
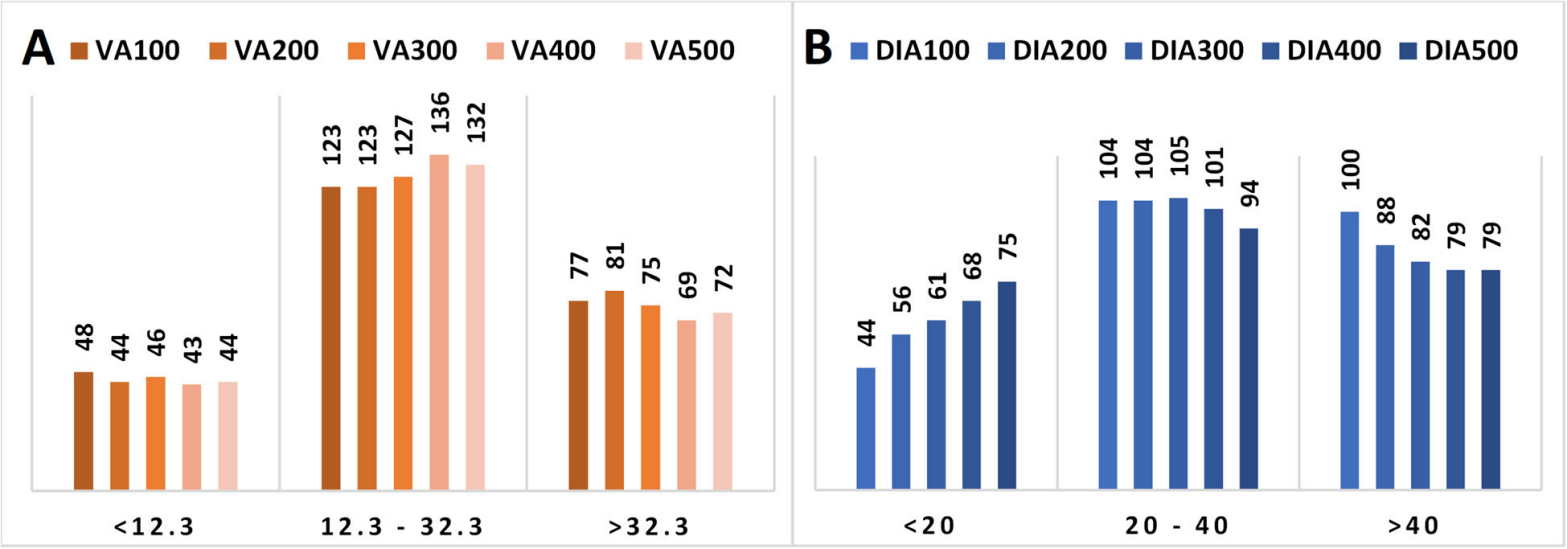
Number of cases in each Ki-67 category (Low, Intermediate and High) for each 100 cell-increment in **A** Visual assessment (VA) and **B** digital image analysis (DIA)

Using the VA median-derived cut-off levels, 48 cases (19.4%) were classified as Ki-67 Low at VA100, falling to 44 (17.7%) at VA500. Twelve cases were upgraded from Ki-67 Low at VA100 to Ki-67 Intermediate at VA500. None were upgraded from Low to High. Similarly, a total of 123 cases (49.6%) were classified as Intermediate at VA100, rising to 132 (53.2%) at VA500. Eight of these cases were downgraded from Intermediate at VA100 to Low at VA500, and eight were upgraded to High. A total of 77 cases (31.4%) were classified as High at VA100 falling to 72 (29.0%) at VA500. Thirteen cases were downgraded from High at VA100 to Intermediate at VA500, and none were downgraded to Low (Fig. 6).

#### Digital image analysis

Using the DIA median-derived cut-off levels, 44 cases (17.7%) were classified as Low at DIA100, rising to 75 (30.2%) at DIA500. Thus, with increasing number of cells counted a further 31 cases (12.5%) were classified as Low. None were upgraded from Low to Intermediate at DIA500. One hundred and four cases (41.9%) were classified as Intermediate at DIA100, falling to 94 cases (37.9%) at DIA500. Thirty cases were downgraded from Intermediate at DIA100 to Low at DIA500. None were upgraded to High. One hundred cases (40.3%) were classified as High at DIA100, falling to 79 (31.9%) at DIA500. Twenty-six cases were downgraded from High at DIA100 to Intermediate at DIA500. None were downgraded from High to Low (Fig. 6).

The numbers of cases classified as Low were similar in VA100 (48 cases), VA500 (44 cases) and DIA100 (44 cases) but increased at DIA500 (75 cases). The number of cases classified as High was greatest at DIA100 (100 cases), falling to levels comparable with VA100 (77 cases) and VA500 (72 cases) at DIA500 (79 cases) (Table 1; Fig. 6).

### Ki-67 and histopathological grade

#### Grade 1

Among the 16 Grade 1 tumours, six (37.5%) tumours were classified as Ki67 Low at VA500. Five cases were classified as Low at DIA100 rising to nine (56.3%) at DIA500 (Table 1).

#### Grade 2

Of the 131 Grade 2 tumours, 13 (9.9%) were classified as High at VA500. Using DIA, 30 (22.9%) were High at DIA100 falling to 21 (16%) at DIA500. A higher number of Grade 2 tumours were classified as Intermediate in VA compared to DIA (Table 1).

#### Grade 3

Of the 101 Grade 3 tumours, 59 (58.4%) were classified as High at VA500. Using DIA, 70 (69.3%) were High at DIA100, falling to 58 (57.4%) at DIA500. The number of Grade 3 tumours classified as Low was greatest at DIA500 (12 (16%)) (Table 1).

### Ki-67 and mitotic count

There was a clear association (*p* < 0.001) between high mitotic count (> 14.5 mitoses/10 HPF) and Ki-67 High across all counting modalities. The highest number of cases were observed at DIA100 where 51 of 62 (82.3%) cases with high mitotic count were classified as Ki-67 High (Table 1).

### Ki-67 and prognosis

There was no clear association between Ki-67 cell counts and risk of death. By the end of follow-up, 108 (43.5%) patients had died of BC.

For VA100 High, the cumulative risk of death from BC during the first five years after diagnosis was 32.5% (95% CI 23.3–44.2), and 46.8% (95% CI 36.4–58.5) 10 years after diagnosis.

**Fig. 7:**
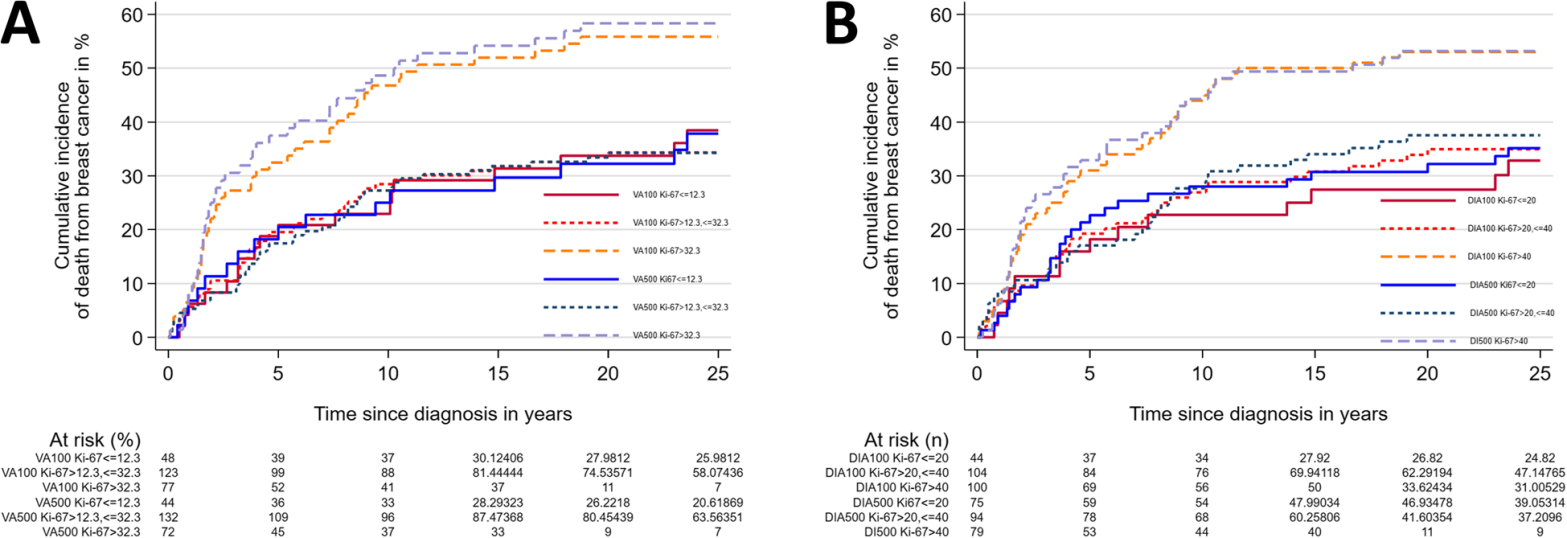
Cumulative incidence of death from breast cancer. **A** Visual assessment (VA), Grays’ test for VA100 (Low, Intermediate and High) *P* = 0.1062; Grays’ test for VA500 (Low, Intermediate and High) *P* = 0.0500. **B** Digital image analysis (DIA), Gray’s test for DIA100 (Low, Intermediate and High) *P* = 0.3335; Grays’ test for DIA500 (Low, Intermediate and High) *P* = 0.0796

For VA500 High, the corresponding risks were 37.5% (95% CI 27.5–49.7) and 48.6% (95% CI 37.8–60.7), respectively. Using VA500 Low as the reference, the rate of death from BC was unchanged for VA500 Intermediate but was higher for VA500 High (HR 1.94 ((95% CI 1.1–3.4))(Table 2; Fig. 7A).

**Table 2:** Risk of death from breast cancer according to Ki-67 level and counting procedures, expressed as cumulative incidence and hazard ratios of death from breast cancer

	**VA100**			**VA500**		
	*Low*	*Intermediate*	*High*	*Low*	*Intermediate*	*High*
Cum. inc. 5 years, % (95% CI)	18.8 (10.2–32.9)	19.5 (13.5–27.7)	32.5 (23.3–44.2)	18.2 (9.5–33.1)	17.4 (11.9–25.1)	37.5 (27.5–49.7)
Cum. inc. 10 years, % (95% CI)	22.9 (13.4–37.6)	28.5 (21.3–37.3)	46.8 (36.4–58.5)	25.0 (14.7–40.6)	27.3 (20.5–35.7)	48.6 (37.8–60.7)
HR (95% CI) ^1^	1.00	1.01 (0.59–1.72)	1.73 (1.01–2.98)	1.00	1.01 (0.58–1.76)	1.94 (1.1–3.42)
Harrell’s C ^1^		0.58			0.59	
h (95% CI) ^2^	1.00	0.92 (0.53–1.60)	1.4 (0.76–2.58)	1.00	0.96 (0.55–1.68)	1.65 (0.85–3.19)
Harrell’s C ^2^	0.60			0.60		
	**DIA100**			**DIA500**		
	*Low*	*Intermediate*	*High*	*Low*	*Intermediate*	*High*
Cum. inc. 5 years, % (95% CI)	15.9 (7.9–30.5)	19.2 (12.9–28.2)	31.0 (22.9–41.1)	21.3 (13.7–32.4)	17.0 (10.8–26.3)	32.9 (23.7–44.4)
Cum. inc. 10 years, % (95% CI)	22.7 (12.9–38.1)	26.9 (19.5–36.6)	44.0 (34.9–54.3)	28.0 (19.3–39.6)	27.7 (19.8–37.9)	44.3 (34.2–55.9)
HR (95% CI) ^1^	1.00	1.14 (0.63–2.06)	1.80 (1.02–3.19)	1.00	1.00 (0.62–1.64)	1.60 (0.99–2.58)
Harrell’s C ^1^		0.58			0.57	
h (95% CI) ^2^	1.00	1.08 (0.59–1.97)	1.48 (0.78–2.82)	1.00	0.93 (0.56–1.53)	1.27 (0.72–2.22)
Harrell’s C ^2^	0.61			0.60		

^1^ Unadjusted. ^2^ Adjusted for tumour grade (1, 2 or 3). *CI* Confidence interval, *Cum. inc.* Cumulative incidence, *HR* Hazard ratio

For DIA100 High, the cumulative risk of death from BC during the first five years after diagnosis was 31.0% (95% CI 22.9–41.1) and after 10 years 44.0% (CI 34.9–54.3).

For DIA500 High, risk was 32.9% (CI 23.7–44.4) within the first five years, and 44.3% (CI 34.2–55.9) within the first 10 years.

Using DIA100 Low as the reference, the rate of death from BC was unchanged for DIA100 Intermediate but was higher for DIA100 High (HR 1.80 (95% CI 1.02–3.19), Table 2; Fig. 7B).

### Comparison of methods

**Fig. 8:**
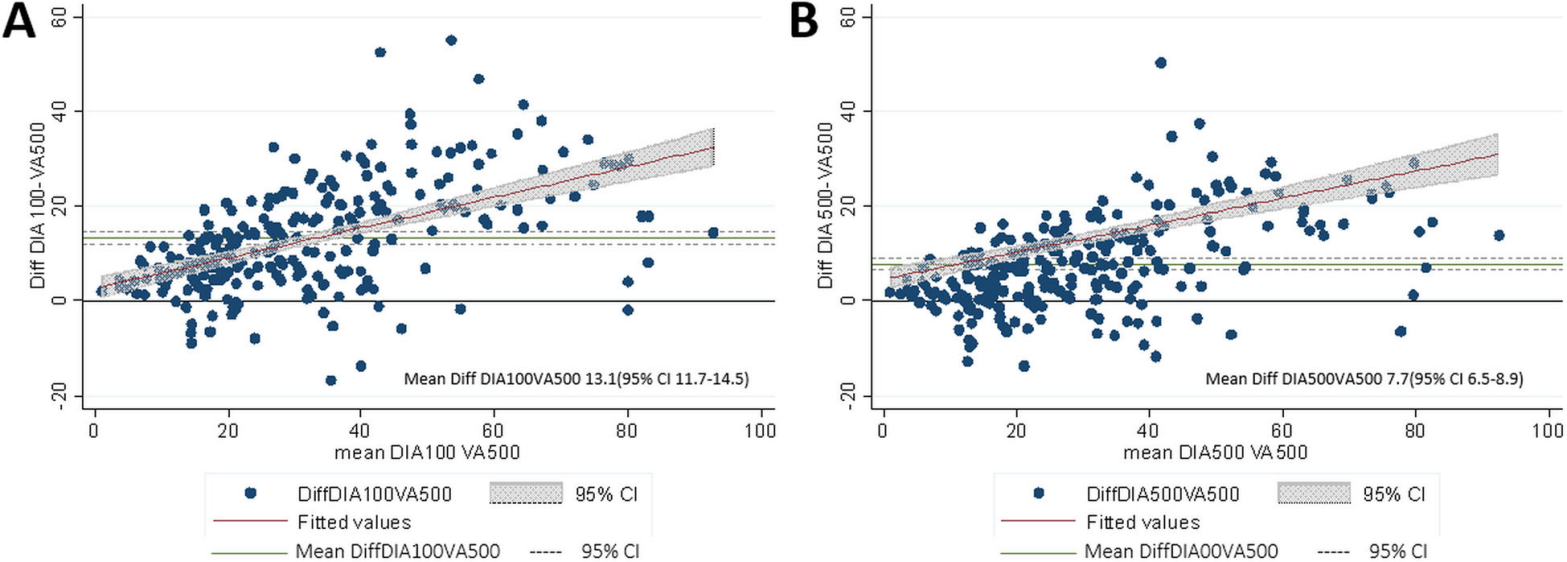
Bland-Altman plots illustrating the agreement of the VA and DIA methods. **A** The difference between DIA100 and VA500 compared to the mean of DIA100 and VA500. **B** The difference between DIA500 and VA500 compared to the mean of VA500 and DIA500

The Bland-Altman plots show that both DIA100 and DIA500 were clearly correlated to VA500. However, the mean values for Ki-67 using DIA (100 and 500) were on average higher than those for VA500, and the differences between DIA and VA500 increased with increasing mean values (Fig. 8). Harrell’s C test showed no clear difference in predictive ability between the VA and DIA methods. A Cox model including grade and DIA100 correctly predicted survival times in 61% of cases, compared to 60% of cases for models combining grade and any one of the other three methods (VA100, VA500 and DIA500).

## Discussion

In this study we compared Ki-67 protein expression in IHC-stained BC tissue sections assessed by DIA using the QuPath platform, and by VA according to current recommended guidelines [22, 23]. We found that the median Ki-67 level was higher using DIA compared to VA. We show that while the proportion of Ki-67 positive tumour cells did not change substantially with increasing number of cells counted using VA, the number of cells counted did impact the result when using DIA. Furthermore, the highest proportion of patients with Ki-67 High tumours was found when 1-200 cells were counted using DIA. All counting methods predicted a poor prognosis for patients with the highest Ki-67 levels, but with little difference between the methods.

Gerdes proposed in 1984 that, with the help of the monoclonal antibody Ki-67, we now had a simple means of estimating the growth fraction of a given subset of human cells. This would be of particular interest in tumour diagnostics since the proportion of proliferating cells in given neoplasms would be of prognostic value and could contribute to the determination of treatment strategies [2]. Ki-67 is now used as a prognostic marker and may also be used as a predictive marker of response to chemotherapy [7–9]. There has been considerable debate regarding counting methods and cut-of levels for both prognostication and determination of treatment [10, 16, 33–37].

At the St. Gallen conference in 2015, it was proposed that the in-house median value at each laboratory should be used to determine cut-off values to offset interlaboratory differences [17]. More recently, the 17th St. Gallen International Breast Cancer Conference proposed that Ki67 should be used to determine treatment in estrogen receptor-negative, HER2-negative T1-2N0-1 BC in accordance with the International Ki67 Breast Cancer Working Group. The determination of cut-off levels is still challenging as reflected by these latest recommendations where only clearly low or clearly high levels of KI67 protein expression are considered to have clinical utility [13, 24]. Romero and co-workers suggested in 2014 a stepwise counting strategy without fixed denominators, especially to target heterogenetic tumours with some highly proliferative hotspots [29] and the International Ki67 Breast Cancer Working Group has proposed a standardized visual scoring method using a scoring app available online [13]. Thus, the need for a standardized approach in the IHC assessment of Ki-67 in BC has been recognized.

In this study, we found clear differences in the median levels of Ki-67 positivity between VA and DIA (VA500 (22.3%) and DIA500 (30%)) reflecting the respective method’s ability to identify hotspot areas in the tissue section. This is in agreement with previous studies [38–41]. Still, others have reported no real differences between the two methods [38, 41–44]. In the present study, the threshold set for OD sum in DIA and thus the ability to digitally detect positive Ki-67 staining, was set close to the pathologist’s threshold for positive staining before commencement of classifier training and digital assessment. The difference between the median values in VA and DIA, suggests that there is need for calibration of cut-off levels according to the method employed. The Bland-Altman plot [45, 46] shows that the methods perform quite similarly but that DIA in general reported higher levels of Ki-67 positivity compared to VA. Introduction of DIA for the assessment of Ki-67 in our hands would thus require recalibration of cut-off levels in order to correspond to established clinically actionable Ki-67 levels. This underlines the importance of understanding the consequences the introduction of a new method may have on patient treatment. However, Harrell’s C test [47] and risk-of-death analyses did not show any clear difference between methods in their ability to predict survival.

Recent studies have suggested that downgrading of Ki-67 levels in some tumors may occur in VA when more than 2-300 cells are counted [29, 48]. However, in the present study we found that there was little difference in the percentage of Ki-67 positive cells in each of the five 100-cell increments across cut-off levels using VA. This would imply that it may not be necessary to count more than 2-300 cells in VA. On the other hand, there was a clear fall in the number of Ki-67 High cases and a corresponding rise in the number of cases classified as Low with increasing cell counts using DIA. Thus, using DIA, the highest proportion of Ki-67 positive cell nuclei is achieved by counting 1-200 cells in digitally identified hotspots. This appears to be in agreement with Romero et al. [29]. In our hands, a significantly higher number of grade 3 tumours was found in DIA100 High compared to VA100 High, VA500 High and DIA500 High (*p* < 0.0001). Thus, we show that declining Ki-67 levels are more likely to occur using DIA compared to VA. A greater number of deaths from BC was seen at DIA100 Ki-67 High compared to DIA500 Ki-67 High (54 vs. 42 cases; 50.0% vs.38.9%). In comparison, for VA, the difference in the numbers of deaths from BC between the VA100 Ki-67 High group and VA500 Ki-67 High group were negligible (43 vs. 42 cases; 40.0% vs. 38.9%).

The cases included in our study were diagnosed with BC over a timespan extending from 1961 to 2008, and pre-analytical conditions may have varied. Ki-67 IHC is robust in formalin-fixed, paraffin-embedded tissue [49, 50] and antigenicity is well preserved, though staining intensity is prone to be reduced with increasing storage-time [51–53]. In the present study, staining intensity was not assessed. The international Ki-67 in Breast Cancer Working Groups has expressed concern about Ki-67 assessment of tissue stored in paraffin-blocks for more than five years, because of the degradation of the epitope in paraffin blocks. The exact mechanisms of the Ki-67 epitope degradation are not yet fully explored and there is still concern about the precision of the assessment. They recommend that the internationally standardized laboratory guidelines (ASCO and CAP) for HER2 and hormone receptors should also be applied to Ki-67 IHC [13]. Variation in tissue processing, staining reagents, laboratory protocols, and digitization procedures, may all contribute to variability in the interpretation of IHC in both conventional VA and DIA. Standardization of the preanalytical and analytical phases of tissue processing would greatly contribute to the creation of a more robust classifier for the digital analysis, although BC’s inherent heterogeneity would still remain a challenge [21, 54, 55]. In the present study, we included only invasive cancer (not otherwise specified). The classifier would require further development to reliably identify tumour cell nuclei morphologies such as those typical of lobular carcinoma. We found that some tissue slides were not suitable for DIA due to artefacts such as tissue folds, damaged tissue, or inadequate staining.

Studies comparing the QuPath platform with other digital analysis platforms have shown good reproducibility and functionality [38, 56]. One study comparing DIA using QuPath with VA shows that QuPath gave stronger prognostic stratification than the manual method [57]. The QuPath software was developed to improve the efficiency, objectivity, and reproducibility of digital histopathology, as well as biomarker analysis using digital images [27]. In the present study a greater number of cases were classified as either Low or High using QuPath DIA compared to conventional VA. Using the Ventana Virtuoso platform, Kwon et al., reported high concordance between VA and DIA, and stronger accuracy using DIA in the High Ki-67-group (≥ 20%) compared to the low Ki-67-group (≤ 10%). They also found that DIA is more useful in the borderline cases between cut-off levels citing observer variation as a greater challenge in these cases [55].

The initial regions of interest on the WSIs were manually delineated using the brush tool in QuPath. This approach was time-consuming, and automatic tissue detection or WSI annotation would be preferable. The first 100-cell increment counted by DIA was visually selected within the area of the tumour with the highest expression of Ki-67 in the heat map. To identify these hotspots, we created measurement maps for nucleus DAB OD mean with 50 μm smoothing. In this process we were aware that tissue folds, ink debris and abundant lymphocytes could result in higher OD in non-relevant areas. Thus, the measurement map method for detecting hotspots may not be suitable in sections with too many such irregularities and artefacts. We noted that membranous staining presented a greater challenge to the QuPath software than to experienced pathologists. A pathologist will ignore non-relevant staining, while the software will detect anything with color, unless the classifier is trained to ignore it.

In the present study, the QuPath-based DIA method entailed a considerable amount of manual adjustment, thus rendering it time-consuming and impractical for implementation in a clinical setting. Robertson et al. published a paper in 2020 that suggested that a digital global scoring of Ki-67 was a practical and clinically valid approach [58]. The International Ki67 in Breast Cancer Working Group discuss several methods including global score and hot spot score in addition to their own online scoring app giving a weighted global score based on the assessment of 100 cells in each of four areas in the tumour section (negligible, low, medium, or high). To the best of our knowledge, the latter has not achieved widespread acceptance. They point out that none of the current scoring systems achieved high analytical validity [13]. Global scoring was not evaluated in the present study. We chose to follow the guidelines for visual assessment of Ki-67 in BC currently in use in Norway, counting 500 cells in the area of the tumour with highest proliferation as assessed under the light microscope [23]. We used the same approach in the digital assessment. We acknowledge that this method may have drawbacks but in comparing the two methods our main finding remains that recalibration of cut-off levels is essential when introducing new methodology in the assessment of tissue biomarkers [23].

The number of cases in this study was limited and thus survival analyses should be interpreted with caution. Our results need to be validated in larger series of cases from other sources. However, the study clearly illustrates that new methodology in biomarker assessment requires recalibration of established cut-off levels.

## Conclusions

In this study we show that assessment of Ki-67 in breast tumours using DIA identifies a greater proportion of cases with high Ki-67 levels compared to VA of the same tumours. Using VA, we found that the results do not change substantially with increasing number of cells counted. However, we propose that, using DIA, it may be sufficient to count 1-200 cells in a digitally selected hotspot area to identify the greatest number of cases with Ki-67 High tumours. Associations with survival should be interpreted with caution due to the limited number of cases and variation of pre-analytical conditions of the tissue samples in this study. Finally, our findings underline the need for recalibration of established cut-off levels on the introduction of new methodology.

## Data Availability

The datasets generated and/or analysed during the current study are not publicly available due to issues of sensitivity and limitations determined in the conditions for approval by the Regional Ethics Committee. However, they can be made available from the corresponding author on reasonable request.

## Abbreviations

BC: Breast cancer
IHC: Immunohistochemistry/immunohistochemical
DIA: Digital image analysis
VA: Visual assessment
OD: Optical density
WSI: Whole slide images
CI: Confidence interval
